# Biodegradation of Phenol at High Initial Concentration by *Rhodococcus opacus* 3D Strain: Biochemical and Genetic Aspects

**DOI:** 10.3390/microorganisms13010205

**Published:** 2025-01-18

**Authors:** Tatiana O. Anokhina, Tatiana Z. Esikova, Valentina N. Polivtseva, Nataliya E. Suzina, Inna P. Solyanikova

**Affiliations:** 1 Laboratory of Plasmid Biology, G.K. Skryabin Institute of Biochemistry and Physiology of Microorganisms, Pushchino Center for Biological Research, Russian Academy of Sciences, Prosp. Nauki 5, 142290 Pushchino, Russia; to_anohina@rambler.ru (T.O.A.); das3534@rambler.ru (T.Z.E.); 2 Laboratory of Cytology of Microorganisms, G.K. Skryabin Institute of Biochemistry and Physiology of Microorganisms, Pushchino Scientific Center for Biological Research, Russian Academy of Sciences, Prosp. Nauki 5, 142290 Pushchino, Russia; v.polivtseva@pbcras.ru (V.N.P.); suzina_nataliya@rambler.ru (N.E.S.); 3 Laboratory of Microbial Enzymology, G.K. Skryabin Institute of Biochemistry and Physiology of Microorganisms, Pushchino Scientific Center for Biological Research, Russian Academy of Sciences, Prosp. Nauki 5, 142290 Pushchino, Russia; 4 Regional Microbiological Center, Institute of Pharmacy, Chemistry and Biology, Belgorod National Research University, 308015 Belgorod, Russia

**Keywords:** *Rhodococcus opacus* strain 3D, phenolic compounds, degradation, free-living cells, immobilized cells, biodegradation enzymes, biodegradation genes, biotechnology

## Abstract

Phenolic compounds are an extensive group of natural and anthropogenic organic substances of the aromatic series containing one or more hydroxyl groups. The main sources of phenols entering the environment are waste from metallurgy and coke plants, enterprises of the leather, furniture, and pulp and paper industries, as well as wastewater from the production of phenol–formaldehyde resins, adhesives, plastics, and pesticides. Among this group of compounds, phenol is the most common environmental pollutant. One of the cheapest and most effective ways to combat phenol pollution is biological purification. However, the inability of bacteria to decompose high concentrations of phenol is a significant limitation. Due to the uncoupling of oxidative phosphorylation, phenol concentrations above 1 g/L are toxic and inhibit cell growth. This article presents data on the biodegradative potential of *Rhodococcus opacus* strain 3D. This strain is capable of decomposing a wide range of toxicants, including phenol. In the present study, cell growth with phenol, growth after rest, growth of immobilized cells before and after rest, phase contrast, and scanning microscopy of immobilized cells on fiber were studied in detail. The free-living and immobilized cells can decompose phenol concentrations up to 1.5 g/L and 2.5 g/L, respectively. The decomposition of the toxicant was catalyzed by the enzymes catechol 1,2-dioxygenase and *cis*,*cis*-muconate cycloisomerase. The role of protocatechuate 3,4-dioxygenase in biodegradative processes is discussed. In this work, it is shown that the immobilized cells can be stored for a long time (up to 2 years) without significant loss of their degradation activity. An assessment of the induction of genes potentially involved in this process was taken. Based on our investigation, we can conclude that this strain can be considered an effective destructor that is capable of degrading phenol at high concentrations, increases its biodegradative potential during immobilization, and retains this ability for a long storage time. Therefore, the strain can be used in biotechnology for the purification of aqueous samples at high concentrations from phenolic contamination.

## 1. Introduction

Phenolic compounds are an extensive group of natural and synthetic organic substances of the aromatic series containing one or more hydroxyl groups. The main sources of phenols entering the environment are waste from metallurgy and coke plants, enterprises of the leather, furniture, and pulp and paper industries, as well as wastewater from the production of phenol–formaldehyde resins, adhesives, plastics, and pesticides [1,2]. Among this group of compounds, phenol is the most common environmental pollutant. Its content in wastewater can vary from several mg to tens of grams/L, depending on the type of product [3,4]. The main risk of phenol is associated with its relatively good solubility in water and the ability to form highly toxic chlorine derivatives that occur during the chlorination of water for the purpose of disinfection. When ingested into reservoirs, phenol inhibits microflora and self-purification processes and is toxic to aquatic organisms, plants, and humans [3].

Various physico-chemical methods, including oxidation, hydrolysis, adsorption-flocculation, solvent extraction, ion exchange purification, reverse osmosis, etc., are known to remove phenols from wastewater [5]. These methods require high economic costs, additional reagents with their subsequent regeneration, and disposal of waste [6,7]. However, microbiological methods of purification of phenolic effluents are cheap, economically profitable, and widely used [8,9,10]. At the same time, the degradation of pollutants occurs without the formation of harmful by-products, which makes it possible to exclude secondary contamination of the environment. The limiting factor for the use of phenol-destructive microorganisms is their inability to utilize high concentrations of phenol and their sensitivity to other toxic compounds present in wastewater.

The processes of biodegradation of phenolic compounds by microorganisms are being actively investigated. Phenol-destructive microorganisms are described among bacteria [11,12], mycelial fungi [13], yeast [14,15], and archaea [16,17]. Phenol-destructive bacterial strains mainly belong to proteobacteria (genera *Pseudomonas*, *Sphingomonas*, *Alcaligenes*, *Comamonas*, *Burkholderia*, *Acinetobacter*, *Ralstonia*, *Cupriavidus*), actinobacteria (*Rhodococcus*, *Arthrobacter*, *Streptomyces*, *Gulosibacter*), and firmicutes (*Bacillus*, *Paenibacillus*) were reported [18,19,20,21,22,23,24].

The conversion of phenol to catechol (the initial intermediate of the central pathways of catabolism of aromatic compounds) by phenol hydroxylase (EC 1.14.13.7) is the first stage of aerobic catabolism of phenol by bacteria. Further cleavage of the aromatic ring of catechol is carried out via two pathways. During *ortho*-cleavage (intradiol), the enzyme catechol 1,2-dioxygenase (Cat 1,2-DO) (EC 1.13.11.1) breaks the ring between two adjacent carbon atoms carrying hydroxyl groups and converts catechol into *cis,cis*-muconic acid, which is further metabolized into succinate and acetyl-CoA in the course of successive reactions. Genes encoding the *ortho*-pathway (β-ketoadipate pathway) of catechol oxidation usually have a chromosomal localization [25]. During *meta*-cleavage (extradiol), splitting of the catechol ring occurs between hydroxylated and non-hydroxylated carbon atoms. The enzyme catechol 2,3-dioxygenase (Cat 2,3-DO) (EC 1.13.11.2) oxidizes catechol to 2-hydroxymucone semialdehyde (2-HMS), which is then converted to acetaldehyde and pyruvate. In most cases, the genes encoding extradiol dioxygenases have plasmid localization [26]. The products of catechol oxidation eventually enter the tricarboxylic acid cycle [25,26,27].

In modern research, much attention is paid to searching for strains capable of utilizing high concentrations of phenol (1000–2000 mg/L) [18,28,29], the study of degradation processes under suboptimal conditions in the presence of other toxic compounds [22,23,30], the use of immobilized cells to increase degradation efficiency [24,31,32] and the creation of stable microbial associations [33,34,35]. A special focus in these studies was actinobacteria of the genus *Rhodococcus*, which are characterized by high ecological plasticity, resistance to stress factors [36,37,38], and the ability to degrade a wide range of natural and synthetic compounds [39,40]. *Rhodococci* have a large genome (about 6.23 Mb on average) with a high (62.3–70.6%) content of guanine-cytosine pairs [18], often carry biodegradation plasmids, and contain many functional analog genes with duplicate functions [40]. For example, in the *R. jostii* RHA1 strain, 26 pathways of peripheral metabolism and 8 pathways of central catabolism of aromatic compounds were identified in the 9.7 MB genome [41]. It has also been shown that the most active phenol destructor strains contain an increased number of copies of genes involved in its degradation [18]. For example, in the strains *R. ruber* C1, *R. jostii* RHA1, *R. aetherivorans* IcdP1, and *R. opacus* B4, the total number of copies of phenol metabolism genes was 50, 51, 51, and 54, respectively.

Previously, we isolated a strain of *R. opacus* 3D that degrades naphthalene through cinnamic acid derivatives, coumarin, and *o*-phthalate [42]. Preliminary experiments have shown that *R. opacus* 3D was capable of growing and utilizing phenol as the only source of carbon and energy. The purpose of this work was to study the possibility of degradation of high concentrations of phenol by the *R. opacus* 3D as well as the effect of cell immobilization and their long-term storage on the destructive activity of the strain. Special emphasis was given to the identification of the biochemical features of degradation and the study of genes involved in the decomposition of phenol in this microorganism.

## 2. Materials and Methods

### 2.1. Chemicals

For the preparation of cultural media, the reagents were obtained from manufacturing companies and meet the requirements of the level of research conducted. Biochemical reagents were obtained from Sigma-Aldrich (St. Louis, MO, USA), Serva (Heidelberg, Germany), Thermo Fischer Scientific (Waltham, MA, USA), Zymo Research (Irvine, CA, USA), New England BioLabs (Hitchin, UK), Biocom (St. Petersburg, Russia), and Fermentas (Vilnius, Lithuania) provided reagents for molecular biology research.

### 2.2. Bacterial Strains Used in the Study

The object of the study was the *Rhodococcus opacus* strain 3D (VKM Ac-2911D), previously isolated from activated sludge from waste treatment facilities (Moscow region, Russia) by direct seeding on an agarized mineral medium with naphthalene as the only source of carbon and energy [42]. Strain 3D was selected for this research based on preliminary data on its ability to utilize a wide range of aromatic compounds, including phenol.

### 2.3. Investigation on the Ability of the Strain R. opacus 3D to Utilize Different Organic Substrates

The strain *R. opacus* 3D was tested for the ability to utilize different aromatic, aliphatic, and chlorinated compounds as a sole source of carbon and energy. For this, the strain *R. opacus* 3D was grown in a mineral salt (MS) medium described previously [42]. Sterile growth substrates were added to the molten agar-containing (1.5% *w*/*v*) medium in the following concentrations (g/L): succinate, naphthalene—0.5–1.0; salicylate, benzoate, gentisate, protocatechuate, catechol, *ortho*-phthalate, 2-hydroxycinnamic, 2-carboxycinnamic acids—0.2–0.5; phenol—0.1–2.5; chlorophenols (2-, 3-chlorophenol, 2,3-, 2,4-, 2,5-, 2,6-dichlorophenol)—0.1; chlorobenzoates (2-, 3-, 4-chlorobenzoate, 3,5-dichlorobenzoate)—0.2. Water-soluble sodium salts of the aromatic acids were used in the experiment.

Volatile aromatic and aliphatic substrates (2-methylnaphthalene, phenanthrene, anthracene, fluorene, acenaphthene, benzene, toluene, ethylbenzene, xylenes (*ortho*-, *meta*-, and *para*-xylene), phenol, hexane, octane, nonane, decane, undecane, dodecane, hexadecane, and coumarin) were applied on a lid of an inverted Petri dish in order to grow cells in vapors of these compounds.

The strain was cultivated at 30 °C on plates or in batch culture on a shaker (180 rpm) in 750 mL Erlenmeyer flasks containing 100 mL of MS medium. The strain was preserved on agar-containing LB medium [43] and in 15% glycerol (*v*/*v*) at –70 °C for subsequent experiments.

### 2.4. Growth of R. opacus 3D Strain in a Liquid Mineral Medium Containing Phenol

To study the strain growth at various concentrations of phenol, the culture was grown in a liquid MS medium containing 50 mg/L of phenol until a late exponential phase. The cells were precipitated by centrifugation (Rotanta 460R, Hettich, Tuttlingen, Germany) for 10 min at 5000 g and 4 °C and resuspended in 5 mL of the same medium. Next, the inoculate prepared in this way was introduced into 100 mL of MS medium to an initial optical density (OD_560_) of 0.15 ± 0.02 optical units. Phenol was added to the medium at concentrations of 0.5, 0.75, 1.0, 1.5, and 1.75 g/L. The intensity of strain growth was evaluated spectrophotometrically (Shimadzu UV-1800, Kyoto, Japan) by light absorption at a wavelength of 560 nm. Samples for measuring the OD, pH of the medium, and the concentration of the substrate were taken for 1–15 days every 2–24 h, depending on the concentration of phenol and the growth rate of the culture. The pH level of the medium during the cultivation of the strain was maintained at 7.0 by adding 10 n NaOH (*w*/*v*). The abiotic control was a sterile MS medium with phenol (0.2 g/L) without inoculum.

### 2.5. Assay of the Degradation of Phenol by Immobilized R. opacus 3D Cells

Polycaproamide fiber was used as a carrier for cell immobilization. The strain was cultivated in 750 mL shake flasks containing 100 mL of MS medium and 2.0 g of carrier (polycaproamide fiber). Cells pregrown in MS medium containing 1.5 g/L of phenol (the maximum concentration of phenol at which free cell growth was observed) were used as an inoculum. The initial OD_560_ was 0.25 ± 0.03 opt. units. The bacterial cells were cultured for 24 h to immobilize cells on the fiber. Then, the substrate was introduced at concentrations of 0.5, 1.0, 1.5, or 2.5 g/L. Samples for determining the phenol content were taken for 5 days every 12–24 h. The abiotic control for assessing the loss of phenol was a sterile MS medium without the addition of an inoculum containing polycaproamide fiber (2.0 g) and phenol (0.2 g/L).

### 2.6. Degradation of Phenol by Immobilized R. opacus 3D Cells After Storage

To study the effect of preserved culture storage on degradable activity, immobilized cells grown in the presence of 2.5 g/L phenol were used as described in Section 2.5. After complete consumption of the substrate, the fiber with immobilized cells was washed with fresh MS medium and stored in 100 mL of medium without the addition of phenol at a temperature of 6 °C for a month. After the expiration of the storage period of the sample of immobilized cells, phenol was added in the concentrations of 0.5 and 2.5 g/L and cultured at 30 °C for 5 days. Samples for measuring the concentration of phenol were taken every 24 h. The abiotic control was a sterile MS medium with phenol (0.5 and 2.5 g/L) and fiber without immobilized bacteria.

After the experiment, the carrier was washed with MS medium and stored in 100 mL of the same medium without phenol for two years at 6 °C. The phenol degradation experiment was repeated as described above.

### 2.7. Determination of Phenol in the Culture Medium

Phenol concentrations in the samples were determined by direct measurement of the absorption spectrum of the medium in the range of 250–290 nm on the Shimadzu UV-1800 device (Kyoto, Japan) using a calibration curve. The curve was plotted using two-fold serial dilutions of an aqueous solution of phenol at an initial concentration of 2.5 g/L. To determine the concentration of phenol in samples, the supernatant (1.5 mL) after cell sedimentation (Rotanta 460R, Hettich, Tuttlingen, Germany) for 10 min at 10,000× *g* and 4 °C was used.

### 2.8. Determination of the Specific Activity of Enzymes

To determine the activity of phenol biodegradation enzymes, cells were grown in a liquid MS medium containing phenol (0.75 g/L) before the beginning of the stationary growth phase. To determine the enzymatic activity in non-induced cells, bacteria were cultured in a liquid MS medium containing 0.5 g/L succinate. Pretreatment and disruption of cells, preparation of cell-free extract, and carrying out enzymatic reactions were performed as described previously [42]. Enzymatic activity was monitored using a UV-1800 spectrophotometer (Shimadzu, Japan).

The activities of Cat 2,3-DO and Cat 1,2-DO were determined by the rate of formation of 2-hydroxymucone semialdehyde (A375, ε = 33,400 M^–1^·cm^–1^) and *cis,cis*-muconate (A260, ε = 16,900 M^–1^ cm^–1^), respectively, as described earlier [44]. The activity of catechol 1,2-dioxygenase when using 4-chlorocatechol as a substrate was determined by the rate of formation of 3-chloromuconate (A260, ε = 12.4 M^–1^ cm^–1^) under the same conditions. The activities of muconate cycloisomerase (MCI) (EC 5.5.1.1) and protocatechuate 3,4-dioxygenase (PC 3,4-DO) (EC 1.13.11.3) were determined by the decrease in cis, *cis*-muconic acid (A260, ε = 16,900 M^–1^ cm^–1^) and protocatechuate (A290, ε 2870 M^–1^ cm^–1^), respectively [45,46].

Specific enzyme activity was expressed as micromoles of consumed substrate or formed product per minute per 1 mg of total bacterial protein. Protein concentration was determined spectrophotometrically by the Bradford protein assay method [47].

### 2.9. PCR Analysis of Target Genes

The oligonucleotide primers used for amplification of the target genes are listed in Table 1. DNA isolation, amplification of gene fragments, separation of reaction products, staining, and visualization of gels were performed as described previously [42].

### 2.10. Microscopy

#### 2.10.1. Light Microscopy

Light microscopy of samples in the phase contrast mode was carried out using a Nikon Eclipse Ci microscope (Nikon, Tokyo, Japan) equipped with a ProgRes SpeedXT camera (Jenoptic, Jena, Germany).

#### 2.10.2. Scanning Electron Microscopy

The cells of the 3D strain were immobilized to fiber samples and grown under various conditions. They were then treated with 1.5% glutaraldehyde in a 0.05 M cacodilate buffer with pH 7.2 (CB) at 4 °C for 1 h and washed three times in the same buffer. Additionally, the samples were fixed in a 1% OsO4 in CB (3 h, 20 °C). After being dehydrated in a series of alcohols with increasing concentrations (30% to 100%, each for 20 min), the samples were incubated in tert-butanol (Sigma-Aldrich, Sigma, St. Louis, MO, USA) three times for 20 min each at 26 °C and then for 12 h at 4 °C.

The samples underwent drying using the JFD-320 Freeze Drying Device (JEOL, Tokyo, Japan), followed by gold dust coating in the JFC-1600 auto fine coater (JEOL, Tokyo, Japan). Subsequently, they were positioned on SEM stubs for analysis using a JSM-6510LV SEM (JEOL, Tokyo, Japan).

### 2.11. Statistical Data Processing

Statistical analysis was performed using Microsoft Excel 2007 (Microsoft, Redmond, Washington, DC, USA). All studies were conducted in three independent experiments.

## 3. Results and Discussion

### 3.1. The Degradation Potential of R. opacus 3D

At the initial stage, the ability of the *R. opacus* 3D strain to grow in an MS medium containing various organic substrates as the only source of carbon and energy was studied (Table 2). As can be seen from the presented data, the studied strain grew in the presence of naphthalene, coumarin, aromatic carboxylic acids (*o*-phthalic, gentisic), benzene and its derivatives (phenol, toluene, ethylbenzene, 2-hydroxycinnamic acid), as well as *n*-alkanes with a carbon chain length of C9–C16, with an even (decane, dodecane, hexadecane) and an odd (nonane, undecane) number of carbon atoms. The culture grew weaker on *n*-alkanes with a shorter chain length (C6–C8), as well as on 2-carboxycinnamic acid, benzoate, and protocatechuate.

Strain 3D did not utilize polycyclic aromatic hydrocarbons: phenanthrene, 2-methylnaphthalene, fluorene, acenaphthene, anthracene, as well as salicylate, which is a key intermediate of naphthalene degradation. The strain also did not grow in MS media containing chlorophenols and chlorobenzoates, which distinguishes it from the well–studied strain *R. opacus* 1CP, a destructor of a number of chlorine-containing compounds, in particular 2,4-dichlorophenol [50]. This can be explained by the fact that strain 1CP was isolated on a medium containing 2,4-dichlorophenol as a carbon source, whereas 3D was selected on a selective medium containing naphthalene. Thus, a wide range of recyclable substrates indicates a significant biodegradative potential of the *R. opacus* 3D strain. Further research was focused on a more detailed study of the phenol degradation process by this strain.

### 3.2. Degradation of Phenol by Strain R. opacus 3D

**Figure 1:**
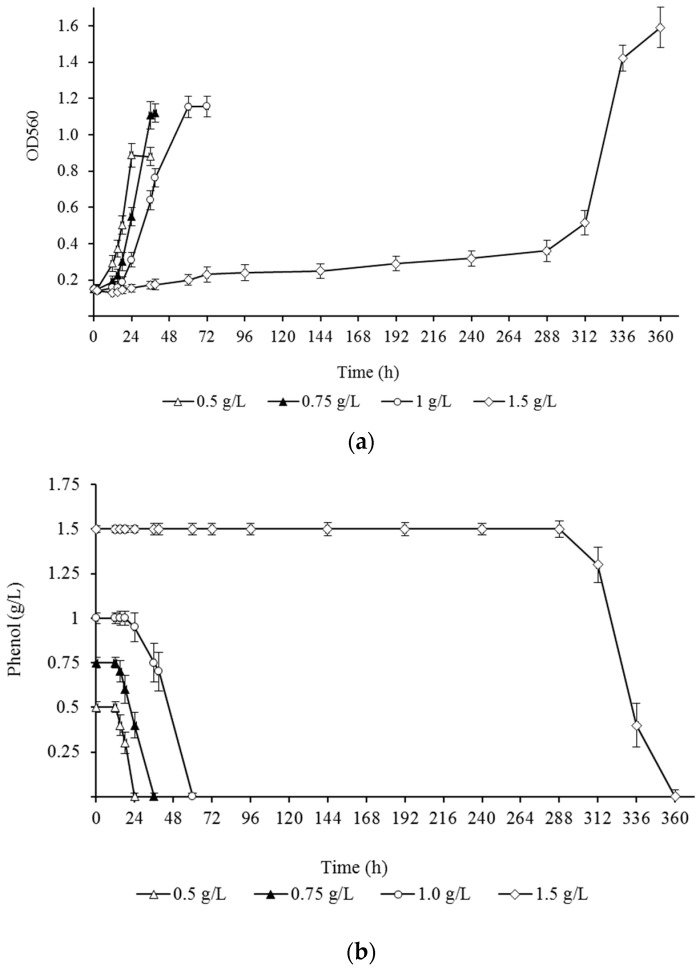
Growth (**a**) and utilization dynamics (**b**) of *R. opacus* 3D in a mineral salt medium at different concentrations of phenol (g/L) as a sole carbon and energy source. Each value is a mean of three replicates with error bars indicating the standard deviation from the mean.

It is known that phenol is a toxic compound that inhibits bacterial growth, including those degrading bacteria, at certain concentrations. High phenol content in wastewater is a critical factor that determines the rate and extent of its degradation. The overwhelming mass of bacteria is sensitive to a relatively low concentration, up to 500 mg/L, of this toxicant. However, single strains capable of decomposing more than 1.5 g/L of this compound have been described [29]. To evaluate the ability of *R. opacus* 3D to utilize high phenol concentrations, we monitored the growth parameters of the culture (duration of the lag phase and OD560) during its cultivation in an MS medium supplemented with 0.5 up to 1.75 g/L substrate. The obtained results are presented in Figure 1a. In the experiments, the duration of the lag phase and the maximum value of the OD depended on the amount of substrate in the medium. The shortest duration of the lag phase (2 h) was observed when cultivating the strain in the presence of 0.5 g/L of phenol. The duration at a substrate concentration of 0.75 and 1.0 was 12 and 18 h, respectively. It should be noted that the growth of the culture at 1.5 g/L of phenol began only after a long adaptation period lasting more than 96 h (Figure 1a).

The growth of the bacterium was accompanied by the consumption of phenol and, accordingly, a decrease in its concentration in the culture medium (Figure 1b). Complete degradation of phenol at an initial concentration of 0.5, 0.75, and 1.0 g/L was carried out in 24, 36, and 60 h, which corresponded to the achievement of the culture of the highest values of OD_560_ in each variant of the experiment (0.89 ± 0.07, 1.11 ± 0.08, and 1.15 ± 0.06, respectively). At 1.5 g/L phenol content, the total consumption of the substrate occurred after a longer period of time (360 h). But at the same time, the culture reached a maximum value of OD560—1.59 ± 0.11 optical units. Cell growth and degradation of phenol at concentrations greater than 1.5 g/L were absent. There was no decrease in phenol concentration in the control variant (without the addition of bacteria).

### 3.3. Degradation of Phenol by Immobilized R. opacus 3D Cells

**Figure 2:**
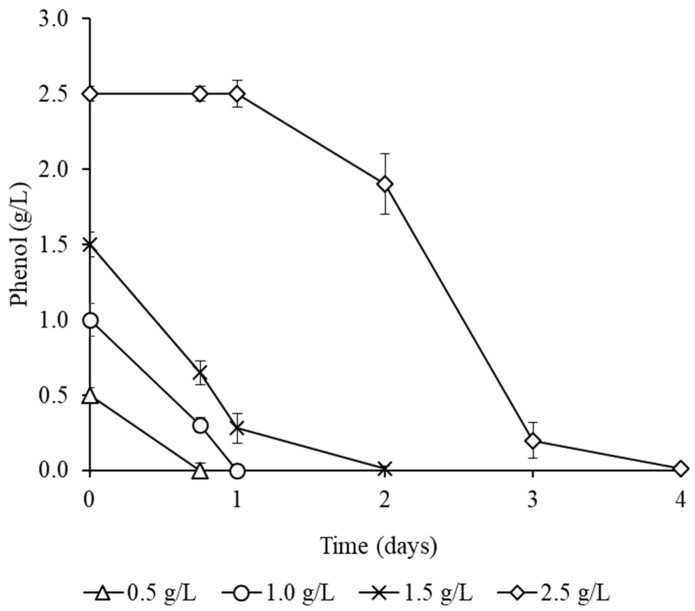
Degradation of phenol by *R. opacus* 3D cells immobilized on a polycaproamide fiber.

Cell immobilization is a widespread phenomenon in nature that ensures the effective survival of bacteria in suboptimal environmental conditions and allows for maintaining a high level of catalytic activity. The immobilization of bacteria on various carriers is widely used in the processes of biodegradation and biotransformation of complex organic substrates, as well as in biotechnology for the production of valuable compounds [51,52,53]. This allows for an increase in the concentration of the target substrate, improves cell survival, and protects them from possible adverse effects. In this regard, the effect of the immobilization of *R. opacus* 3D cells on polycaproamide fiber on the efficiency of phenol degradation, especially those presented at high concentrations, was studied. The maximum phenol content in the experiment was 2.5 g/L. Cells adapted to high concentrations of phenol were used as an inoculum. To do this, they were cultured until the end of the logarithmic growth phase in a mineral medium containing 1.5 g/L of phenol. As can be seen from the data presented in Figure 2, the studied strain completely disposed of 1.0, 1.5, and 2.5 g/L of phenol in 1, 2, and 4 days, respectively. At the same time, a significant reduction in the duration of the lag period was observed. Thus, the immobilization of cells of the strain–destructor led to a significant reduction in the degradation time of the substrate compared with free cells (Figure 1b). For example, the utilization time of 1.5 g/L of phenol was reduced from 15 to 2 days. In addition, during cell immobilization, the maximum concentration of phenol, at which culture growth was maintained, was increased to 2.5 g/L. The immobilization of cells probably contributed to their resistance to extremely high concentrations of toxic substrate and their ability to recycle it. A similar effect has been described for other strains–destructors. For example, vermiculite-immobilized cells of *R. aetherivorans* UCM Ac-602 utilized 2.0 g/L of phenol in 96 h, whereas free-suspended cells of this strain were unable to grow at this substrate concentration [29]. Immobilization of *Pseudomonas* sp. NBM11 cells on Ca-alginate beads resulted in a 3–3.5-fold increase in the rate of phenol degradation. Thus, complete degradation of phenol (1.0 g/L) by free cells occurred in 168 h and by immobilized cells in 48 h [24]. In our case, cell immobilization led not only to a significant increase in the concentration of phenol but also to the rate of its destruction compared to the described strains. It should be noted that during the experiments, no sorption of phenol by polycaproamide fiber was detected, which confirms the role of the studied strain *R. opacus* 3D in the decomposition of the toxicant.

### 3.4. Degradation of Phenol by R. opacus 3D Strain After Storage of Immobilized Cells

**Figure 3:**
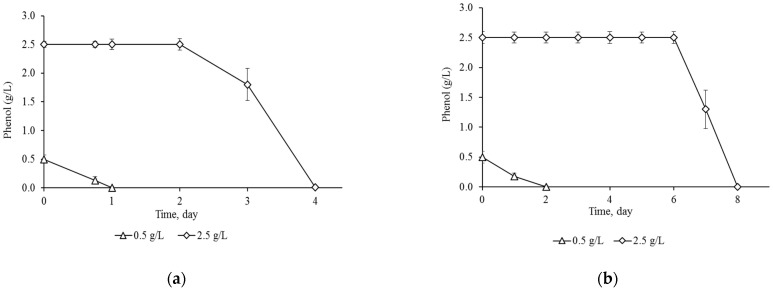
Degradation of phenol by immobilized *R. opacus* 3D cells, after storage at 6 °C for 1 month (**a**) and 24 months (**b**).

It is known that immobilization provides attached cells with increased resistance to negative environmental conditions, high operational stability, and reuse [53,54]. Long-term preservation of culture properties during storage is a key success factor for subsequent use of an immobilized cell system. After storing immobilized *R. opacus* 3D cells in MS medium in the absence of a substrate for 1 month (Section 2.6), the strain completely degraded 0.5 and 2.5 g/L of phenol in 1 and 4 days, respectively (Figure 3a). Consequently, the degradation time of the substrate by cells subjected to a state of rest has not changed compared to cells that have not been stored. The literature describes strains in which the ability to degrade the pollutant was completely preserved or slightly decreased after storage of immobilized cells for 30 days [32,55]. However, a further increase in storage time led to a significant loss of degradation activity of encapsulated *Pseudomonas oleovorans* strain ICTN13 cells [53]. Thus, the catalytic activity of encapsulated cells stored for 30 and 60 days decreased from 53% to 14%, respectively.

Experimental data on the assessment of the effect of a long period of rest (more than a year) on the ability of strains–destructors to decompose pollutants are not available in the literature. In this work, after storage for 2 years, it was shown for the first time that immobilized *R. opacus* 3D cells retained viability, although the rate of phenol degradation significantly decreased. Thus, the studied strain completely disposed of 0.5 g/L of phenol in 2 days, which is about 2.5 times slower than when using immobilized cells that have not been stored. At a concentration of 2.5 g/L of phenol, the duration of degradation of the substrate by stored immobilized cells doubled and reached 8 days. Nevertheless, the cells retained the ability to decompose phenol at high concentrations, which is generally rare for bacterial strains. The obtained results indicated that the *R. opacus* strain 3D compares favorably with the previously described phenol destructor bacteria, since in addition to the ability to utilize high concentrations of phenol, it retains its degradable activity after long-term storage.

### 3.5. Enzymatic Activity in the Bacterial Culture R. opacus 3D Grown in the Presence of Phenol

It is known that the key enzyme for the decomposition of phenol under aerobic conditions is phenol hydroxylase, which transforms the initial substrate into its dihydroxyaromatic derivative—catechol. The opening of the aromatic ring of catechol can be carried out in two ways, in the *ortho*- (1,2) or *meta*-(2,3) position with the participation of Cat 1,2-DO and Cat 2,3-DO, respectively [11,20]. Both pathways are widely represented in the bacterial degradation of phenol. However, an analysis of the literature data shows that the pathway of *ortho*-cleavage of catechol prevails in rhodococci. Thus, it can be assumed that the presence of Cat 1,2-DO activity in cells growing in the presence of phenol is an important condition for the realization of destructive activity by rhodococci against this toxicant.

Catechol dioxygenases are inducible enzymes. The specific activity of enzymes involved in the degradation of phenol in strain 3D was determined both in induced (strain growth in a mineral medium in the presence of phenol) and in non-induced cells (strain growth in the presence of succinate) (Table 3).

In cell-free extracts of the strain grown with phenol, the activity of Cat 2,3-DO was extremely low (0.001 U/mg of protein), while the activities of Cat 1,2-DO and muconate cycloisomerase were significant and amounted to 0.115 ± 0.006 and 0.025 ± 0.007 U/mg of protein, respectively. This indicates that the cleavage of the catechol ring in the studied strain occurs via the *ortho*-cleavage pathway. When using 4-chlorocatechol as a substrate, the specific activity of Cat 1,2-DO was less than 8% of the activity of this enzyme with catechol, which is generally characteristic of catechol 1,2-dioxygenases.

It is known from the literature that the degradation of catechol can be carried out both separately via the *ortho*- or *meta*-pathway and via both biochemical pathways in parallel [56]. For example, the simultaneous activity of catechol 1,2- and 2,3-dioxygenases was recorded in *Planococcus* sp. strain S5 when it was grown at low phenol concentrations (1–2 mM); when the phenol content increased to 3–4 mM, only the activity of Cat 2,3-DO was detected [56]. In our case, when cultivating the 3D strain in the presence of a high concentration of phenol (0.75 g/L), the situation was the opposite; only the activity of Cat 1,2-DO was defined in the cell-free extract.

PC 3,4-DO is a representative of the superfamily of intradiol dioxygenases and catalyzes the *ortho*-cleavage of the protocatechuate ring to form β-carboxy-*cis*,*cis*-muconate [25]. Although this enzyme is not involved in phenol/catechol catabolism, its specific activity in the 3D strain was more than three times higher than the activity of Cat 1,2-DO and amounted to 0.385 ± 0.061 U/mg of protein. The simultaneous presence of Cat 1,2-DO and PC 3,4-DO activity has been described in a number of bacterial strains when grown with various aromatic substrates. For example, in *Pseudomonas putida* strain KT2442, when cultured with *para*-hydroxybenzoate (PHB) and vanillin [57], *Acinetobacter baumanii* DU202 with PHB [58], and *Rhodococcus* sp. RHA1 with benzoate and phthalate [59]. In some cases, there was a significant activity on the excess level of PCT 3,4-DO over Cat 1,2-DO. Thus, when *Pseudomonas* sp. 13BN and *Rhodococcus* sp. 7B strains were cultured on phenol (0.5 g/L), and the activity of PC 3,4-DO exceeded the activity of Cat 1,2-DO by more than an order of magnitude [60]. One possible explanation for this fact is the formation of intermediates of catechol catabolism, which induce the synthesis of PC 3,4-DO. It is known that the metabolic reactions of *ortho*-oxidation of catechol and protocatechuate are two branches of the β-ketoadipate degradation pathway of aromatic compounds in microorganisms [25]. The key intermediate of this pathway, β-ketoadipate, serves as an inducer of the synthesis of enzymes involved in the oxidation of protocatechuate. For example, in *Nocardia*, a bacterium phylogenetically related to *Rhodococcus*, β-ketoadipate induces the synthesis of almost all enzymes of the protocatechuate branch, including PC 3,4-DO [61]. Perhaps in our case, as in the above examples, a similar phenomenon is observed.

When the studied strain was cultivated in the presence of succinate, i.e., non-induced by phenol cells, the specific activity of the enzymes Cat 1,2-DO, MCI, and PC 3,4-DO was significantly lower when compared to the activity of cells grown in the presence of phenol, which indicates the inducible nature of their synthesis (Table 3). It should be noted that the activity level of PC 3,4-DO (0.021 ± 0.003 U/mg of protein) under non-selective conditions was also higher compared to Cat 1,2-DO (0.006 ± 0.003 U/mg of protein). The activity of Cat 2,3-DO on succinate was extremely low and similar to the activity of cells grown with phenol. This fact suggests that Cat 2,3-DO is not involved in phenol metabolism.

### 3.6. PCR Analysis of Rhodococcus opacus 3D Genome

**Figure 4:**
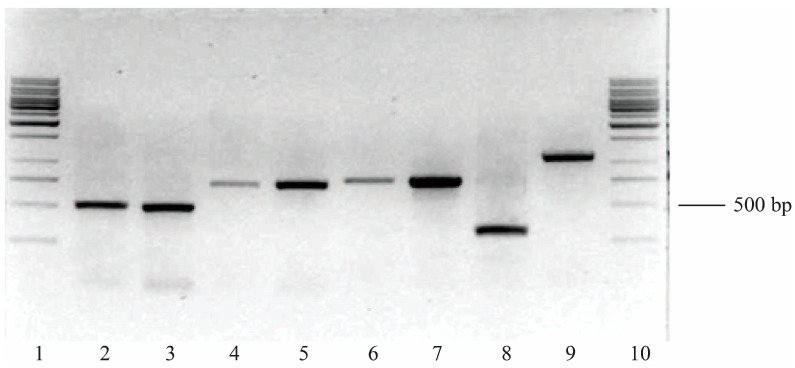
Screening of *R. opacus* 3D for genes encoding protocatechuate 3,4-dioxygenase (*pcaH*), catechol 1,2-dioxygenases (*catA*, *catA2*), and catechol 2,3-dioxygenases (*edoB* and *edoC*). The strain *R. opacus* 1CP was used as a positive control. Lanes: 1, 10—molecular weight marker, 1 kb DNA Ladder (Fermentas, Lithuania); 2, 3—*pcaH* from *R. opacus* 3D (2) and 1CP (3); 4, 5—*catA2* from *R. opacus* 3D (4) and 1CP (5); 6, 7—*catA* from *R. opacus* 3D (6) and 1CP (7); 8—*edoB* from *R. opacus* 3D; 9—*edoC* from *R. opacus* 3D.

To identify phenol degradation genes in the 3D strain, we used specific primers developed on the basis of a sequence of isofunctional genes Cat 1,2-DO (*catA*, *catA2*), PC 3,4-DO (*pcaH*) of the *R. opacus* 1CP strain [49], and genes of extradiol dioxygenases (*edoB*, *edoC*) of *Rhodococcus rhodochrous* strains P200 and *Rhodococcus* sp. I1 [48]. The results of the amplification of the genes encoding Cat 1,2-DO, Cat 2,3-DO, and PC 3,4-DO are shown in Figure 4. In the *R. opacus* 3D strain, a positive response was obtained when using all primers. The size of the amplified products corresponded to the expected one and did not differ from the control strain of *R. opacus* 1CP. A positive response with primers developed for the extradiol dioxygenase genes was obtained only for the 3D strain, unlike *R. opacus* 1CP, in which a PCR product of the appropriate size was not detected.

The presence of the *catA* and *catA2* genes and the high activity of the Cat 1,2-DO enzyme indicate that phenol utilization in this strain occurs via the *ortho*-pathway of oxidation of its key intermediate, catechol. The presence of extradiol dioxygenase genes usually indicates that catechol utilization can occur via a *meta*-pathway. However, in our case, the low activity of Cat 2,3-DO in the 3D strain indicates that the genes of extradiol dioxygenases are not expressed when growing with phenol. Earlier [42], the studied strain also showed low activity of Cat 2,3-DO when growing in the presence of naphthalene. It is possible that in this bacterium, the genes of the catechol *meta*-cleavage pathway are not functioning (silent genes), or they are expressed during the utilization of other aromatic substrates, for example, methylated aromatic hydrocarbons such as toluene and ethylbenzene.

When cultivating the culture in a mineral medium in the presence of succinate in a cell-free extract, a sufficiently high (0.021 ± 0.003 U/mg protein) activity of PC 3,4-DO was detected. The presence of the *pcaH* gene and the high activity of PC 3,4-DO may indicate that the synthesis of this enzyme is constitutive. When growing strain 3D in an MS medium with phenol, the activity of PC 3,4-DO in the cell-free extract increased by more than an order of magnitude compared with the mineral medium + succinate variant (Table 3). It is possible that the metabolites formed during the oxidation of phenol are non-specific inducers of the synthesis of this enzyme, the role of which in the catabolism of phenol is not yet clear. The confirmation of the role of PC 3,4-DO in the degradation of phenol at the concentration of 1500 mg/L after long adaptation is shown in the work of Ma and co-authors [62]. Using the method of transcriptomic analysis, the authors showed that at the initial stages of phenol utilization by the *Burkholderia* sp. strain, activation occurred not only of the genes of the catechol branches of the β-ketoadipate pathway but also of the protocatechuate one, although after a day the transcriptional activity of the PC 3,4-DO coding gene decreased. Unfortunately, this work lacks data on the relative activity of Cat 1,2-DO and PC 3,4-DO in cells, which helped expand the understanding of the biodegradation of toxic pollutants by bacteria.

Thus, the amplification of intradiol dioxygenase genes (*catA*, *catA2*, *pcaH*), the high activity of the corresponding enzymes Cat 1,2-DO and PC 3,4-DO, and the ability of the strain to grow with phenol and protocatechuate indicate the functioning of the catechol and protocatechuate branches of the β-ketoadipate degradation pathway of aromatic compounds in strain 3D.

### 3.7. Morphology of R. opacus 3D Free-Living Cells

**Figure 5:**
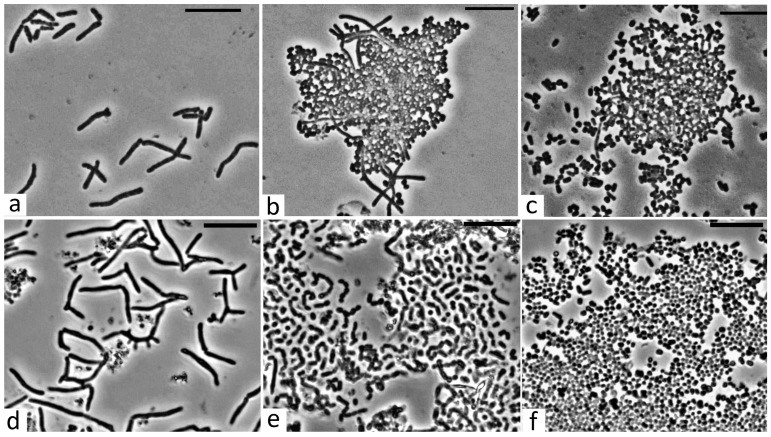
Morphology of *R. opacus* 3D cells under various cultivation conditions. (**a**) growth with succinate (1.0 g/L), day 1; (**b**) growth with succinate (1.0 g/L), day 2; (**c**) growth with succinate (1.0 g/L), day 7; (**d**) growth with phenol (1.0 g/L), 1st day; (**e**,**f**) growth with phenol, day 2, 36 and 48 h. Light microscopy. Phase contrast. Scale bar—10 µm.

Previously, it was shown that rhodococci are characterized by pleomorphism; their distinctive feature is a three-stage morphogenetic cycle of development (rod-shaped—filamentous or branching cells—cocci) [38]. Nevertheless, the study of transitions between cell forms has shown that it is largely determined by the nature of the growth substrate. When growing with succinate (Figure 5a), the population of *R. opacus* 3D cells is represented by uneven, long sticks of irregular, slightly curved shape. The cell length under these conditions is mainly in the range from 3 to 5 microns, sometimes (rarely) reaching ~10 microns; the cell diameter is ~0.7–0.8 microns. The process of transition of a population from a rod-shaped cell shape has taken quite a long time. On the second day of growth, the cells of the 3D strain began to split (Figure 5b), and cocci up to 1 micron in diameter started to appear, but only a part of the population cells were affected by this process. The entire process took about a week, and almost the whole population consisted of cells in the form of cocci (Figure 5c).

Cells germinating in an MS medium with phenol as a growth substrate behaved identically to cells germinating in a growth medium with a non-toxic substrate; however, the rate of morphometric changes was significantly different. When cells grew in an MS medium with phenol (1.0 g/L), a significant elongation of the cell population occurred in the phase of logarithmic growth, which was accompanied by the formation of elongated branching cell forms (Figure 5d). The cell length in these conditions reached 15 microns with a cell thickness of ~0.7–0.8 microns. However, this process took only the first day. After a day and a half, synchronous fragmentation of these cell forms occurred (Figure 5e), and under these conditions, by the end of the second day, the cell population was almost completely represented by cocci with a cell diameter of 0.7–0.8 microns (Figure 5f). This behavior of cells in the presence of a toxic substrate has been shown for the first time.

### 3.8. Morphology of Immobilized R. opacus 3D Cells

**Figure 6:**
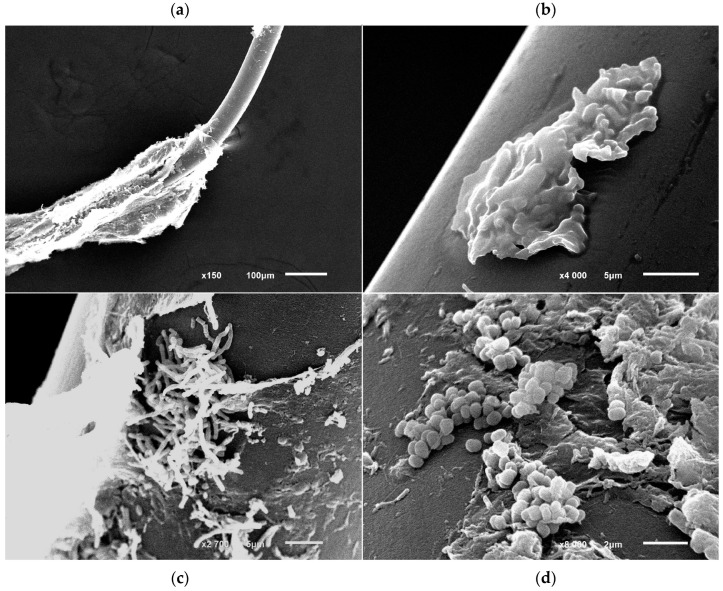
Fiber with *R. opacus* 3D cells immobilized on it when cultured in a liquid mineral medium in the presence of phenol (1.0 g/L). Scanning electron microscopy. (**a**,**b**) are clusters of cells immersed in the intercellular matrix; (**c**) are clusters of rod-shaped cells in the logarithmic growth phase; (**d**) are coccoid cells in the stationary growth phase. Scale bar: 100 µm (**a**), 5 µm (**b**,**c**) and 2 µm (**d**).

The analysis obtained from scanning microscopy on the immobilized cells of *R. opacus* 3D on fiber images showed that the fouling (immobilization) of cells on the fiber surface occurs discretely in the form of cell clusters and is accompanied by the formation of an intercellular matrix in the form of layers of films and fibrils, which ensures the connection of cells with each other and with the fiber surface (Figure 6a,b). Perhaps the matrix also has a protective function, since it often covers entire groups of cells. Clusters of immobilized cells differ in size and number of cells contained in them. Single immobilized cells are rare. Such cells have the form of extended rods with formed septa and non-separated daughter cells (incomplete stage of division).

The morphology of immobilized *R. opacus* 3D cells was similar to the morphology of free cells grown with succinate in a liquid medium during periodic cultivation. Cells in clusters were represented by either rod-shaped or coccoid shapes. Probably, in each particular cluster, the cells are at a different stage of development (have a different physiological status). Clusters with long rod-shaped cells are probably in the logarithmic growth phase (Figure 6c), and coccoid cells are in the stationary phase (Figure 6d). Most likely, each type of cluster was started by a single cell, which was at one stage or another of growth at the time of immobilization. Given the relatively large number of rod-shaped cells, it can be concluded that the protective effect of immobilization largely minimized the toxic effects of phenol.

Thus, the behavior of immobilized cells, even in the presence of a toxic substrate, resembles the growth of cells on a non-toxic substrate with transitions between rod-shaped and coccoid cell forms. The reason for this may be the protective matrix formed by cells, which neutralizes the negative effects of a toxic substrate. In addition, it is known that cells in the attached state are more resistant to the effects of negative factors compared to free-living ones. This was clearly demonstrated by the example of hydrocarbon-oxidizing strains of bacteria of the genus *Rhodococcus*, for which a direct relationship between adhesive and hydrocarbon-oxidizing activities was shown [63]. Attached *Rhodococcus* cells retain viability and high metabolic activity for a long time.

## 4. Conclusions

The *Rhodococcus opacus* 3D strain studied in this work was characterized by the ability to decompose a wide range of toxicants, including alkanes (nonane, decane, undecane, dodecane, hexadecane), phenol, benzene, toluene, ethylbenzene, naphthalene, 2-hydroxycinamic acid, coumarin, phthalate, and gentisate. It was previously established that the destruction of naphthalene by this strain does not imply the formation of salicylate, which is considered a “classic” intermediate during bacterial decomposition. In this work, the process of phenol destruction was investigated for both free-living and immobilized cells. As expected, catechol 1,2-dioxygenase and *cis*,*cis*-muconate cycloisomerase have been identified as the main enzymes of phenol degradation by this strain. However, high activity of protocatechuate 3,4-dioxygenase was found in both non-induced and phenol-induced cells, the role of which in the decomposition of phenol by this strain remains unclear. A distinctive feature of *R. opacus* 3D cell culture is the ability to decompose phenol in a concentration of up to 1.5 g/L and 2.5 g/L by non-immobilized and immobilized cells, respectively. Moreover, high cell survival has been shown for this strain. This was expressed in the ability to resume metabolic activity and decomposition of phenol after storage of immobilized cells for 2 years. This ability allows us to store prepared biomaterial for the effective removal of phenol from effluents, for example, in the case of emergent bioremediation.

## Figures and Tables

**Table 1:** Target genes and PCR primers used in this study.

Putative Gene Function (Gene)	Primer	Primer Sequence (5’–3’)	Annealing Temp (°C)	Amplicon Size (bp)	Reference
Catechol 2,3-dioxygenase (*edoB*)	LK33 LK38	CGCATCGAGGCCACCGAC CGACGGGTCCTCGAACGTGA	61	306	[48]
Catechol 2,3-dioxygenase (*edoC*)	VD11 VD14	GGTTACATGGGCTTCGAG CTCCGCCGACTTCTCCAG	58	1096	-//-
Protocatechuate 3,4-dioxygenasde (*pcaH*)	pcaH_260f pcaH_749r	CAACGCCGACATCGCCAA GAATCCGACGGCCCAGTTGT	54	489	[49]
Catechol 1,2-dioxygenase (*catA2*, GenBank FM877593.1)	cat4f cat4r	AAATTCAAGGGCGCAAG GAGTTCGGGTTTCGTTG	52	720	-//-
Catechol 1,2-dioxygenase (*catA*, GenBank X99622.2)	cat6f cat6r	CGACAAGTTCAAGGCCGAG CACGAAGTTGTAGGTGACGTAG	52	780	-//-

**Table 2:** Spectrum of substrates utilized by strain *R. opacus* 3D.

Substrate	Growth *
Alkanes (nonane, decane, undecane, dodecane, hexadecane) Phenol, benzene, toluene, ethylbenzene Naphthalene, 2-hydroxycinnamic acid, coumarin Phthalate, gentisate	Good growth
Alkanes (hexane, heptane, octane) Benzoate, protocatechuate 2-carboxycinnamic acid	Weak growth
PAHs (phenanthrene, 2-methylnaphthalene, fluorene, acenaphthene, anthracene) Salicylate *Ortho*-, *meta*-, *para*-xylene Chlorophenols (2-, 3-chlorophenol, 2,3-, 2,4-, 2,5-, 2,6-, dichlorophenol) Chlorobenzoates (2-, 3-, 4-chlorobenzoate, 3,5-dichlorobenzoate)	No growth

* Bacterial growth was assessed after 3–7 days of cultivation.

**Table 3:** Enzymatic activity in the *R. opacus* strain 3D cells, grown in the mineral medium in the presence of phenol and succinate.

Enzymes	Substrate	Specific Activity, U/mg of Protein, After Growth with	
		Phenol	Succinate ^1^
Cat 1,2-DO	Catechol	0.115 ± 0.006	0.006 ± 0.003
	4-Chlorocatechol	0.009 ± 0.002	n.d.
Cat 2,3-DO	Catechol	0.001 ± 0.000	0.002 ± 0.000
MCI	cis,cis-muconate	0.025 ± 0.007	< 0.001
PC 3,4-DO	Protocatechuate	0.385 ± 0.061	0.021 ± 0.003

n.d.—was not determined, Cat 1,2-DO—catechol 1,2-dioxygenase, Cat 2,3-DO—catechol 2,3-dioxygenase, PC 3,4-DO—protocatechuate 3,4-dioxygenase, MCI—muconate cycloisomerase. ^1^ The specific activities of biodegradation enzymes during cultivation of strain 3D in a mineral medium in the presence of succinate are given from [42].

## Data Availability

Dataset available on request from the authors.
